# The use of PROMIS measures in clinical studies in patients with inflammatory arthritis: a systematic review

**DOI:** 10.1007/s11136-023-03422-0

**Published:** 2023-04-27

**Authors:** M. M. H. Teuwen, I. R. E. Knaapen, T. P. M. Vliet Vlieland, J. W. Schoones, C. H. M. van den Ende, S. F. E. van Weely, M. G. J. Gademan

**Affiliations:** 1 Department of Orthopaedics, Rehabilitation and Physical Therapy, Leiden University Medical Center (LUMC), Albinusdreef 2, P.O.Box 9600, 2300 RC Leiden, The Netherlands; 2 Directorate of Research Policy, Leiden University Medical Center, Leiden, The Netherlands; 3 Department of Research, Sint Maartenskliniek, Nijmegen, The Netherlands; 4 Department of Rheumatology, Radboud University Medical Center, Nijmegen, The Netherlands; 5 Department of Clinical Epidemiology, Leiden University Medical Center, Leiden, The Netherlands

**Keywords:** Systematic review, PROMIS, Patient-reported outcome measures, Rheumatoid arthritis, Axial spondyloarthritis

## Abstract

**Purpose:**

Although the use of Patient-Reported Outcomes Measurement Information System (PROMIS) measures is widely advocated, little is known on their use in patients with inflammatory arthritis. We systematically describe the use and outcomes of PROMIS measures in clinical studies involving people with rheumatoid arthritis (RA) or axial spondyloarthritis (axSpA).

**Methods:**

A systematic review was conducted according to the PRISMA guidelines. Through a systematic search of nine electronic databases, clinical studies including patients with RA or axSpA and reporting the use of PROMIS measure were selected. Study characteristics, details of PROMIS measures and their outcomes, if available, were extracted.

**Results:**

In total, 29 studies described in 40 articles met the inclusion criteria, of which 25 studies included RA patients, three studies included axSpA patients and one study included both RA and axSpA patients. The use of two general PROMIS measures (PROMIS Global Health, PROMIS-29) and 13 different domain-specific PROMIS measures was reported, of which the PROMIS Pain Interference (*n* = 17), Physical Function (*n* = 14), Fatigue (*n* = 13), and Depression (*n* = 12) measures were most frequently used. Twenty-one studies reported their results in terms of *T*-scores. Most *T*-scores were worse than the general population mean, indicating impairments of health status. Eight studies did not report actual data but rather measurement properties of the PROMIS measures.

**Conclusion:**

There was considerable variety regarding the different PROMIS measures used, with the PROMIS Pain interference, Physical function, Fatigue, and Depression measures being the most frequently used. In order to facilitate the comparisons across studies, more standardization of the selection of PROMIS measures is needed.

**Supplementary Information:**

The online version contains supplementary material available at 10.1007/s11136-023-03422-0.

## Plain English summary

Apart from clinical, laboratory tests, or imaging, patient-reported outcome measures (PROMs) are essential to evaluate the outcomes of inflammatory arthritis and its management. With the Patient-Reported Outcomes Measurement Information System (PROMIS), PROMs can be measured in a uniform and standardized way. PROMIS covers specific and generic health domains and are relevant for various patient populations. Specific PROMIS measures such as Physical Function, Fatigue, Sleep Disturbance, or Depression can be used to measure a more specific health domain than the general measures such as PROMIS Global Health. Although the use of PROMIS measures is widely advocated, little is known on their actual use in patients with inflammatory arthritis. In this systematic literature review, we wanted to describe the use and outcomes of PROMIS measures in clinical studies involving people with rheumatoid arthritis or axial spondyloarthritis. This systematic literature review found that PROMIS measures are currently not often used in clinical studies in these patient groups and that there is a large variety regarding the use of specific PROMIS measures. To facilitate the comparisons of outcomes across studies, more standardization of the use of specific PROMIS measures is needed.

## Introduction

Rheumatoid arthritis (RA) and axial spondyloarthritis (axSpA) are two forms of inflammatory arthritis that can lead to pain, stiffness, fatigue, limitations in functioning, and participation in a considerable proportion of patients, despite the availability of effective drug treatments [1–3]. It is beyond doubt that this has a major impact on the quality of life of these patients [1–3].

Apart from clinical, laboratory, or imaging parameters, patient-reported outcome measures (PROMs) are essential to evaluate the outcomes of inflammatory arthritis and its management. To date, numerous PROMs, either generic or disease-specific, are used in clinical care and research regarding inflammatory arthritis. However, some of the widely used legacy measures that are based on the classical test theory are criticized for a lack of standardization, precision, and/or comparability of scores across studies and diseases [4, 5]. To overcome these limitations, in 2007, the Patient-Reported Outcomes Measurement Information System (PROMIS) became available [6]. PROMIS measures are item-response theory-based questionnaires (Item Banks, Short Forms or Computer Adaptive Tests) that cover specific and generic health domains and are relevant for various patient populations. All PROMIS measures use a standardized metric, called a T-score, centered around the general population, which enhances the interpretability of these scores.

PROMIS measures have been applied in general populations and in people with different physical conditions such as critical illness, spinal surgery, low back pain, cancer, and chronic pain [7–12]. For inflammatory arthritis patients, the use of PROMIS measures seems to be appropriate as well, where several PROMIS measures are used since its introduction in 2007. Recently, the International Consortium for Health Outcomes Measurement (ICHOM) promoted the use of PROMIS Pain Interference, General Health, Physical Function, and Fatigue measures as part of routine outcome measurement for patients with inflammatory arthritis [13]. This more standardized way of reporting PROMIS outcomes facilitates new options to compare the performance of health care for inflammatory arthritis on a global scale, allowing health care professionals to learn from each other and to further improve the health care for inflammatory arthritis patients.

Little is known so far about the extent and nature of their actual use in clinical research in patients with inflammatory arthritis. Thus, the aim of this review was to systematically determine the use and outcomes of PROMIS measures in clinical studies including patients with RA and/or axSpA. The outcomes of PROMIS measures were included to assess whether the PROMIS measures depict the relatively worse health status of RA and axSpA patients.

## Methods

### Study design

This systematic review was reported according to the Preferred Reporting Items for Systematic Reviews and Meta-Analyses (PRISMA) [14], with the exception of the PRISMA item on risk of bias assessments, as the methodological quality of the studies was deemed less relevant given the exploratory nature of the literature review.

### Search strategy

A trained librarian (JS) performed a literature search in nine electronic databases (PubMed, MEDLINE, Embase, Web of Science, Cochrane Library, Emcare, PsycINFO, Academic Search Premier, Google Scholar) on July 29, 2022. The search strategy consisted of the combination of the disease concepts (RA, AxSpA and inflammatory arthritis) with PROMIS. Not only controlled subject terms such as MeSH terms were applied, but also various free text words describing the search concepts were used. The search was limited to articles published from 2007 onwards, as PROMIS became available in that year. The search strategy is presented in Supplement 1. The identified records were imported into a software application (Rayyan (http://rayyan.qcri.org) [15] and duplicates were removed. In addition, studies were identified through an indirect approach by screening the references of included studies and those of relevant systematic reviews resulting from the search.

### Selection criteria

- *Inclusion criteria*: Original clinical studies (a) reporting the use of one or more PROMIS measures; (b) including patients with RA and/or axSpA aged 18 years or above; (c) written in English, French, German or Dutch.
- *Exclusion criteria*: Studies including patients with multiple diagnoses, but not reporting the information on RA or axSpA patients separately.

No limitations were formulated on the type of study design (e.g., retrospective studies, prospective studies, randomized controlled trials).

### Selection process

Records retrieved from the search were screened in two phases. In the first phase, all identified records were screened by checking the title and abstract by two researchers (MT, IK) according to the abovementioned eligibility criteria using the online Rayyan® software [15]. Records were scored as most likely eligible, possibly eligible and not eligible. Records that were scored as not eligible were excluded. Disagreements were resolved by discussion between the two researchers and if no agreement was found the record was deemed as eligible for the second phase of screening. Subsequently, 10% of all records in the first phase (title and abstracts) were screened by a third researcher (TVV) to ensure the quality of the selection process.

In the second phase, full-text articles were retrieved and independently screened by the same two researchers, using the same eligibility criteria. For that purpose, the outcomes of the screening were entered into a pre-defined database with the inclusion and exclusion criteria. Disagreements were resolved by discussion between the two researchers and if no agreement was found, a third researcher was consulted (TVV or MG). Fifty percent of the screening of the full-text papers was checked by a third researcher (MG). The third reviewer was a supervisor (TVV /MG), who was engaged to further ensure the quality of the screening process. For feasibility reasons, given the total amount of titles and abstracts versus full-text papers, 10% and 50% of the selection and extraction processes was checked.

### Data extraction

A pre-defined data extraction form was used to systematically extract information from the full-text articles that were ultimately selected. One researcher extracted the data (MT or IK), a second researcher checked this extraction (MT or IK). Again, a third researcher checked the data extraction of 50% of the papers to ensure the data were correctly extracted (MG).

Regarding the study characteristics, information on the first author, year of publication, country, study design (cross-sectional study, longitudinal cohort study, controlled or uncontrolled clinical trial, other; based on definition of the original study) and population (registry, community or clinic) was retrieved. With respect to the study populations we collected: type of inflammatory arthritis (RA, axSpA or both), the number of patients, general patient characteristics (mean age, sex, disease duration).

We defined articles as individual papers unless the data of two or more articles were gathered in the same community, clinic(s) or registry, and the sample sizes and general patient characteristics (age, sex distribution) were exactly the same, in that case we addressed these articles as one single study. The date of the first publication was used for the chronological ordering of the studies. However, if one of these publications included T-scores and the other publications did not, the date of the publication reporting on T-scores was used.

The name of the PROMIS measures (Item Banks, Short Forms, Computer Adaptive Tests) used with version number was recorded and checked with the website of *healthmeasures.net*, accessed on August 1, 2022. If the name of the reported PROMIS measure was not registered, the measure was not taken into account. Also the results of T-score metrics were extracted, if available. For T-scores a normalized distribution (T-score 0–100, standardized mean 50, standard deviation 10) is used. A value of 50 is considered as the mean score of the general population with a standard deviation of 10. For some PROMIS measures a score higher than 50 indicates a better outcome (e.g., PROMIS Ability to Participate in Social roles and Activities, Physical Function, Satisfaction with Social roles and Activities), whereas for others a score higher than 50 means a worse outcome (e.g., Anger, Anxiety, Fatigue, Pain Behavior, Pain Intensity, Pain Interference, Sleep Disturbance, Sleep-Related Impairment, Depression). If a PROMIS measure was administered multiple times in one study and likewise reported, the results at baseline were extracted.

If the results of a specific PROMIS measure were reported in multiple articles that were grouped in one study, and there was a difference between T-scores smaller than 0.5, the score reported in the first publication was extracted. In case of any scores that were unclear, the first author of the article was contacted, to confirm the calculation.

## Results

**Fig. 1:**
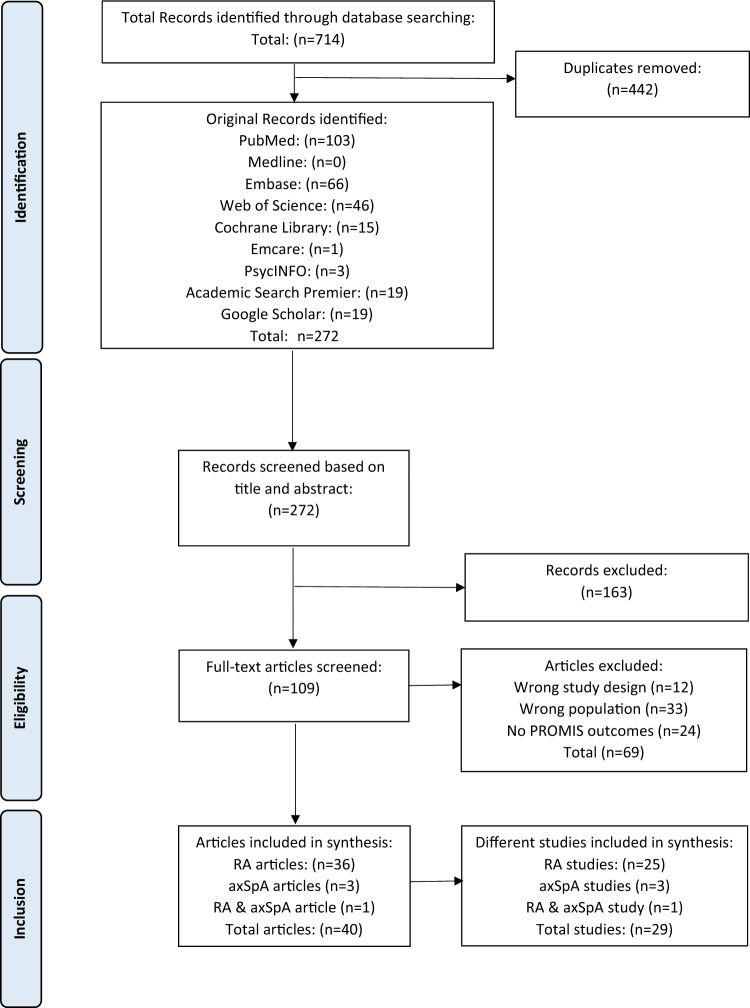
Flowchart of screening process

The search identified initially 714 records, which after deduplication resulted in a set of 272 records. The first screening resulted in the exclusion of 163 records (Fig. 1). After the screening of the remaining 109 full-text articles, 69 were excluded. Thus, in total, 40 articles were included [16–55], reporting on 29 studies, including 25 studies in RA patients three studies in axSpA patients and one study on both RA and axSpA patients. The flow of the screening process is shown in Fig. 1.

The publication years of the studies ranged from 2011 up to 2022, with relatively more studies being published in recent years. Of the 29 studies, three studies were published in 2011–2015, 13 studies in 2015–2019 and 13 studies in 2020-present.

The characteristics of the 29 included studies (total number of 22,855 patients) are summarized in Table 1. Overall, most of the studies originated from the US (23 of 29 studies; 79.3%). The study designs included cross-sectional studies (10 of 29 studies, 34.5%), longitudinal cohort studies (15 of 29 studies; 51.7%), randomized controlled trials (two of 29 studies, 6.9%), or other (one pilot study 3.4% and one cross-over study, 3.4%).

**Table 1:** Characteristics and used PROMIS measures of clinical studies in patients with axSpA and RA

Study	Country	1 CS 2 LC 3 RCT 4 Other	1 RA 2 axSpA	N	Female (%)	Population	Age Mean (SD)	Years since diagnosis mean (SD)	General measures		PROMIS measures of a specific domain										
									GH	P-29	Physical function	Fatigue	Pain IF	Pain BH	Pain IT	Sleep Disturbance/Sleep-Related Impairment	Satisfaction with Social Roles and Activities	APS	Anxiety /anger	Depression	SEMS
Gavigan et al. 2022 [55]	USA	2	2	195	87.0	Registry: Arthritis Power Registry	47.6(10.6)	38.6(11.2)^a^			×		×			×					
Becker et al. 2021 [16]	USA	1	1	20 12	65.0 67.0	Community: used Pain interference Community: used Sleep Disturbance	53.2 (11.4) 55.0 (8.5)	9.2 (7.4) 13.3 (12.0)					×			×					
Bingham et al. 2021 [17]	USA	2	1	11	91.0	Clinic: Johns Hopkins Arthritis Centre	55.0 (12.0)	20.0 (10.0)				×	×								
Cano-García et al. 2021^e^ [18]	SP	1	1 2	50 51	90.0 51.0	Clinic: RA patients Clinic: axSpA patients	55.1 (13.6) 52.5 (12.1)	14.3 (7.1) 13.0 (6.1)										×			
Lee et al. 2021 [19]	USA	2	1	3949	83.0	Registry: The National Databank for Rheumatic Diseases (FORWARD) registry	65.4 (11.9)	21.7 (12.6)					×								
Craig et al. 2020^f^ [20–22]	USA	2	1	196 262	81.0 82.0	Clinic: Johns Hopkins Arthritis Centre Clinics: Johns Hopkins, The Hospital for Special Surgery, University of Alabama	54.8 (13.4) 56.6 (13.9)	11.0 (10.0) 13.2 (11.4)			×	×	×			×		×	×	×	
Gavigan et al. 2020 [23]	USA	1	1	249	92.0	Registry: Arthritis Power Registry	51.7 (11.0)	11.0 (9.5)			×	×	×			×	×				
Heisler et al. 2020 [24]	USA	2	1	263	81.8	Clinics: five US academic medical centers	54.7 (13.8)	9.8 (11.9)	x				×			×			×	×	
Hitchon et al. 2020 [25]	USA	1	1	150	84.7	Clinic & Community: Arthritis Center in Winnipeg	59.8 (11.7)	41.7 (14.9)^a^											×	×	
Hwang et al. 2020 [26]	USA	2	2	119	69.0 M	Clinic: University of Texas Health Science Center	50.9 (14.77)	25.5 (13.3)	×		×	×	×		×					×	
Katz et al. 2020 [27]	USA	2	1	3848	83.1	Registry: The National Databank for Rheumatic Diseases (FORWARD) registry	64.9 (12.0)	20.8 (12.7)					×								
Liew et al. 2020 [28]	USA	2	2	203	66.0 M	Clinic: University of California, San Francisco	46.4 (12.5)	22.9 (12.4)											×		
Prodinger et al. 2020 [29]	Ger UK	1	1	180 535	91.4 74.2	Clinic: sample from Germany Clinic: Sample From UK	49.0 (13.8) 68.3 (10.0)	13.5 (12.2) 19.8 (13.0)			×										
Bingham et al. 2019 [30, 31]	USA	1	1	348	81.0	Clinics: Johns Hopkins, The Hospital for Special Surgery, University of Alabama	57.0 (14.0)	14.0 (11.0)		×	×		×					×		×	
Crins et al. 2019 [32, 33]	NL	1	1	2029 1554	69.0 69.0	Clinic: Outpatient patients from Reade and Leuven, used Pain Interference Clinic: Outpatient patients from Reade and Leuven, used Pain behavior	59.0 (13.0) 59.0 (12.0)	1–12 months 2% 1–2 years 7% 2–5 years 16% > 5 years 75%^c^ 1–12 months 2% 1–2 years 6% 2–5 years 15% > 5 years 76%^c^					×	×							
Izadi et al. 2019 [34]	USA	2	1	595	83.0	Clinic: University of California, San Francisco	56.8 (15.3)				×										
Mahmoud et al. 2019 [35]	EG	1	1	120	94.0	Clinic: Outpatient clinic Cairo University	41.5 (11.1)	6.3 (7.3)			×		×		×	×			×	×	
Wohlfahrt et al. 2019 [36]	USA	2	1	156	82.1	Clinics: five US academic medical centers	54.6 (13.6)	10.0 (12.6)	x			×	×	×	×	×			×	×	
Alleva et al. 2018 [37]	UK/NL	3	1	84	100.0	Community: Female RA population	44.8 (12.5)	11.3 (10.9)											×	×	
Bartlett et al. 2018^ g^ [30, 38]	USA	1	1	200 52 32	84.0 87.0 72.0	Community: Creakyjoints.org Clinic: Johns Hopkins Arthritis Centre patients Clinics: Johns Hopkins, Hospital for Special Surgery, University of Alabama	51.0 (12.0) 53.0 (14.0) 54.0 (13.0)	10.0 (10.0) 15.0 (11.0) 13.0 (10.0)		×	×	×	×					×		×	
Katz et al. 2018 [39]	USA	3	1	96	84.0	Clinic: University of California, San Francisco	54.8 (13.4)	14.8 (12.3)				×	×								
Mollard et al. 2018 [40]	USA	4	1	63		Clinic: Rheumatology and Arthritis Investigational Network Database	> 18 years^d^														×
Bacalao et al. 2017 [41]	USA	2	1	119	91.0	Clinic: Northwestern University Feinberg School of Medicine	57.0(21.0–77.0)^b^	11.0(0–52)^b^			×	×	×							×	
Katz et al. 2017 [42]	USA	2	1	4346	83.0	Registry: The National Databank for Rheumatic Diseases (FORWARD) registry	64.0 (12.0)	20.0 (13.0)		×											
Wahl et al. 2017 [43]	USA	2	1	326	81.6	Clinic: University of California, San Francisco	59.0 (14.0)	12.0(5.0–22.0)^b^			×										
Askew et al. 2016 [44–47]	USA	2	1	521	81.0	Registry: The Arthtritis Rheumatism and Aging Medical Information System (ARAMIS) registry	88% ≥ 50^c^				×	×	×								
Bjorner et al. 2014 [48]	USA	4	1	223	35.0 M	Community: contacted RA patients	56.0 (10.0)				×	×								×	
Oude Voshaar et al. 2014 [49–51]	NL	2	1	690 557	63.6 52.6	Registry: The Dutch Rheumatoid Arthritis Monitoring (DREAM) Community: contacted US RA sample	56.8 (11.8) 56.7 (10.9)				×										
Fries et al. 2011 [52–54]	USA	2	1	451	81	Registry: The Arthtritis Rheumatism and Aging Medical Informatio System (ARAMIS) registry	65				×	×								×	
N articles axSpA patients									1	0	2	1	2	0	1	1	0	1	1	1	0
N studies axSpA patients									1	0	2	1	2	0	1	1	0	1	1	1	0
N articles RA patients									2	3	20	15	18	3	2	8	1	6	8	13	1
N studies RA patients									2	3	12	12	15	2	2	6	1	4	6	11	1

*APS* Ability to Participate in Social Roles and Activities; *axSpA* axial spondyloarthritis; *CS* cross-sectional study; *EG* Egypt; *GH* Global Health; *LC* longitudinal cohort study; *M* male participants; *NL* Netherlands; *N* number; *Pain BH* pain behavior; *Pain IT* pain intensity; *Pain IF* pain interference; *P-29* PROMIS-29; *RA* rheumatoid arthritis; *RCT* randomized controlled trial; *SEMS* Self-efficacy Managing Symptoms; *SP* Spain; *SD* standard deviation; *UK* United Kingdom; *USA* United States of America

^a^Age at diagnosis

^b^Median (range)

^c^Mean and median not available: a percentage of the total sample with a value

^d^Mean and median and percentage not available: value based on inclusion criteria

^e^This study reported on one axSpA population and one RA population. In both populations the same PROMIS measure: Ability to Participate in Social roles and Activities (APS) was used and was therefore counted as one study in the RA patients group and as one study in the axSpA patients group

^f^Craig et al. 2020 describes 2 populations, one population, describing 196 patients, was included in Bartlett et al. 2020a and Direnzo et al. 2020, the other population describing 262 patients was only included in Craig et al. 2020

^g^Bingham et al. 2019 describes 2 populations, one population, describing 348 patients, was included in Bartlett et al. 2020b and one population of 200 was included in Bartlett et al. 2018. The populations of 52 and 32 patients were only included in Bartlett et al. 2018

Table 1 shows the various PROMIS measures identified in the included studies. In total, 17 different PROMIS measures were identified in this review, consisting of two general health measures (PROMIS Global Health and PROMIS-29) and 13 measures pertaining to a specific health domain. The latter included the PROMIS Physical Function, Fatigue, Pain Interference, Pain Intensity, Pain Behavior, Sleep Disturbance, Sleep-Related Impairment, Satisfaction with Social Roles and Activities, Ability to Participate in Social roles and Activities, Anxiety, Anger, Depression, and Self-Efficacy Managing Symptoms. The four most frequently used measures were: PROMIS Pain Interference (17 studies), Physical Function (14 studies), Fatigue (13 studies), and Depression (12 studies).

Table 2 shows the details of the specific versions of PROMIS measures being used, classified according to their typology into Item Banks, Computer Adaptive Tests (CATs), and Short Forms. Some of the variation regarding the versions can be explained by the publication dates, with more recent articles reporting more recent versions of a similar PROMIS instrument. Other sources of variation include the precise naming and the number of items used.

**Table 2:** PROMIS measure versions used in axSpA and RA populations

PROMIS measure used	N of articles	N of studies	Publication year	Reference of articles
PROMIS Global Health (GH)	3	3		[24, 26, 36]
PROMIS Global Health Short Form^c^	1	1	2021	[24]
PROMIS Global Health Short Form version 1.1^a^	1	1	2021	[26]
PROMIS Global Health version 1.1^a^	1	1	2019	[36]
PROMIS-29	3	3		[30, 31, 42]
PROMIS Adult profile-29a^c^	2	2	2019, 2017	[30, 42]
PROMIS Adult profile-29 (v2.0)^a^	1	1	2020	[31]
PROMIS Physical Function	22	14		[20–23, 26, 29–31, 34, 35, 41, 43, 46–55]
PROMIS Physical Function^c^	1	1	2021	[46]
PROMIS physical Function Item Bank^c^	1	1	2014	[49]
PROMIS CAT Physical Function^c^	5	4	2022, 2020, 2020, 2020, 2017	[20, 22, 23, 41, 55]
PROMIS CAT Physical Function 10-item	1	1	2014	[50]
PROMIS CAT Physical Function (v.1.0)	1	1	2020	[21]
PROMIS Physical Function 4-items Short Form	1	1	2020	[29]
PROMIS Physical Function 6-items Short Form	1	1	2020	[29]
PROMIS Physical Function 8-items Short Form	2	2	2014, 2011	[48, 52]
PROMIS Physical Function 10-items Short form	3	3	2020, 2015, 2011	[29, 51, 53]
PROMIS Physical Function 10a Short Form	3	3	2019, 2019, 2017	[34, 35, 43]
PROMIS Physical Function 12a Short Form (v1.0)^b^	1	1	2020	[26]
PROMIS Physical Function 20-items Short Form	4	3	2020, 2016, 2011, 2015	[29, 47, 53, 54]
PROMIS Physical Function 20a Short Form	1	1	2019	[30]
PROMIS Physical Function 20a Short Form (v 1.0)^a^	2	2	2020, 2020	[21, 31]
PROMIS fatigue	16	13		[17, 20–23, 26, 30, 31, 35, 36, 38, 39, 41, 45, 46, 48]
PROMIS Item Bank Fatigue^c^	1	1	2021	[17]
PROMIS CAT Fatigue^c^	5	4	2020, 2020, 2020, 2019, 2017	[20, 22, 23, 36, 41]
PROMIS CAT Fatigue (v.1.0)	1	1	2020	[21]
PROMIS Fatigue Short Form (version 1)	2	2	2020, 2016	[26, 45]
PROMIS Fatigue 4a Short Form	1	1	2019	[35]
PROMIS Fatigue 7a Short Form	2	2	2019, 2018	[30, 39]
PROMIS Fatigue 7a Short Form (v 1.0)	4	4	2020, 2020, 2018, 2021	[21, 31, 38, 46]
PROMIS Fatigue 8-items Short Form	1	1	2014	[48]
PROMIS Fatigue 8a Short Form	1	1	2019	[30]
PROMIS Fatigue 8a Short Form (v 1.0)	2	2	2020, 2018	[31, 38]
PROMIS Pain Interference [Pain IF)	20	17		[16, 17, 19–24, 27, 30–33, 35, 36, 39, 41, 44, 46, 55]
PROMIS Pain Interference Item Bank^c^	4	4	2021, 2021, 2021, 2016	[16, 17, 19, 44]
PROMIS Pain Interference Item Bank (v 1.1)^a^	2	1	2019, 2020	[32, 33]
PROMIS CAT Pain Interference^c^	7	6	2022, 2020, 2020, 2020, 2020, 2019, 2017	[20, 22–24, 36, 41, 55]
PROMIS CAT Pain Interference (v1.0)	1	1	2020	[21]
PROMIS Pain Interference 4a Short Form	1	1	2019	[35]
PROMIS Pain Interference 4-items Short Form	1	1	2020	[27]
PROMIS Pain Interference 8-items Short form	1	1	2018	[39]
PROMIS Pain Interference 8a Short Form	1	1	2019	[30]
PROMIS Pain Interference 8a Short Form (v 1.0)^a^	3	3	2020, 2020, 2020	[21, 26, 31]
PROMIS Pain Interference 6b-item Short Form (version 1.1)^a^	1	1	2021	[46]
PROMIS Pain Behavior (Pain BH)	3	2		[32, 33, 36]
PROMIS Pain Behavior Item Bank (v.1.1)^a^	2	1	2019, 2020	[32, 33]
PROMIS CAT Pain Behavior^c^	1	1	2019	[36]
PROMIS Pain Intensity (Pain IT)	3	3		[26, 35, 36]
PROMIS Pain Intensity Short Form (v1.0)	1	1	2020	[26]
PROMIS Pain Intensity 3a Short Form	2	2	2019, 2019	[35, 36]
PROMIS Sleep Disturbance/Sleep-Related Impairment	9	7		[16, 20–24, 35, 36, 55]
PROMIS Sleep Disturbance Item Bank (v1.0)	1	1	2021	[16]
PROMIS CAT Sleep Disturbance^c^	5	4	2020, 2020, 2020, 2020, 2019	[20, 22–24, 36]
PROMIS CAT Sleep Disturbance (v.1.0)	1	1	2020	[21]
PROMIS CAT Sleep-Related Impairment^c^	1	1	2019	[36]
PROMIS CAT Sleep Interference^b^	2	2	2022, 2020	[20, 55]
PROMIS Sleep Disturbance 4a Short Form	1	1	2019	[35]
PROMIS Sleep Disturbance 4a Short Form (v.1.0)	1	1	2020	[21]
PROMIS Satisfaction with Social Roles and Activities	1	1		[20]
PROMIS CAT Satisfaction with Social Roles^c^	1	1	2020	[20]
PROMIS Ability to Participate in Social roles and Activities (APS)	6	4		[18, 20–22, 30, 31]
PROMIS CAT Participation in Social Roles and Activities^b^	1	1	2020	[20]
PROMIS CAT Ability to Participate in Social Participation^b^	1	1	2020	[22]
PROMIS CAT Ability to Participate in Social Roles (v2.0)^b^	1	1	2020	[21]
PROMIS Ability to Participate in Social Roles and Activities 8a Short Form (v.2.0)	3	3	2021, 2020, 2020	[18, 21, 31]
PROMIS Participation in Social Roles and Activities 8a Short Form	1	1	2019	[30]
PROMIS anxiety/anger	9	7		[20–22, 24, 25, 28, 35–37]
PROMIS Anxiety Short Form^b^	1	1	2018	[37]
PROMIS Anxiety Short Form 4a	1	1	2019	[35]
PROMIS Anxiety 4a Short Form (v.1.0)^a^	1	1	2020	[21]
PROMIS Anxiety Short Form (6-items)	1	1	2020	[28]
PROMIS Anxiety 8a Short Form	1	1	2020	[25]
PROMIS CAT Anxiety^c^	4	3	2020, 2020, 2020, 2019	[20, 22, 24, 36]
PROMIS CAT Anxiety (v1.0)^c^	1	1	2020	[21]
PROMIS CAT Anger^c^	1	1	2020	[20]
PROMIS Depression	14	12		[20–22, 24–26, 30, 35–37, 40, 41, 48, 52]
PROMIS Item Bank Depression^c^	1	1	2014	[48]
PROMIS Emotional Distress-Depression Item Bank^c^	1	1	2011	[52]
PROMIS CAT Depression^c^	5	4	2020, 2020, 2020, 2019, 2017	[20, 22, 24, 36, 41]
PROMIS CAT Depression (v1.0)^c^	1	1	2020	[21]
PROMIS Depression Short Form^c^	1	1	2018	[37]
PROMIS Depression 4a Short Form	1	1	2019	[35]
PROMIS Depression 8a Short Form	2	2	2020, 2019	[25, 30]
PROMIS Depression 8a Short Form (v.1.0)	1	1	2020	[21]
PROMIS Emotional Distress-Depression Short Form (v1.0)	1	1	2020	[26]
PROMIS Self-Efficacy Managing Symptoms (SEMS)	1	1		[40]
PROMIS Self-Efficacy Managing Symptoms (P-SEMS)^c^	1	1	2018	[40]

*axSpA* axial spondyloarthritis; *CAT* Computer Adaptive Test; *N* number;* Publication year* If more than one publication year was reported the order was based on the references starting with the most recent publication; *RA* rheumatoid arthritis

^a^Version other than reported on website https://www.healthmeasures.net/ [accessed 1 August 2022)

^b^Name of the instrument is different from the one reported on the website https://www.healthmeasures.net/ [accessed 1 August 2022)

^c^Description is unclear, instrument cannot be linked to a specific PROMIS instrument

In Table 3 the *T*-scores of the PROMIS measures are presented, classified according to the health domain which they represent. In total, eight of the 29 studies did not report actual outcomes of PROMIS measures in terms of *T*-scores, but reported on their psychometric properties (e.g., the validity, reliability, correlations with other questionnaires, responsiveness, meaningful change) only. The 26 articles presenting actual PROMIS data described the results from 21 studies.

**Table 3:** T-scores (SD) of PROMIS measures in populations with axSpA and RA

Study and articles within the study reporting T-scores	Articles or cohorts within the study Reporting PROMIS outcome	GH	P-29	Physical Function	Fatigue	Pain IF	Pain BH	Pain IT	Sleep Disturbance/Sleep-Related Impairment^i^	Satisfaction with social roles and actitvities	APS	Anxiety	Depression
Gavigan et al. 2022 [55]				36.6(6.4)		65.8 (6.2)			60.6 (8.2)				
Cano-García et al. 2021 [18]	RA population[18] axSpA population [18]										RA: 26.2 (7.8) axSpA: 26.9 (8.2)		
Craig et al. 2020 [20–22]	Craig et al. cohort 1 [21] Craig et al. cohort 2 [21]			Cohort 1:43.7 (8.9) Cohort 2: 40.5 (9.7)	Cohort 1: 53.6 (10.1) Cohort 2: 55.0 (10.7)	Cohort 1: 53.5 (9.3) Cohort 2: 57.6 (10.1)			Cohort 1: 51.5 (9.7) Cohort 2: 52.0 (10.5)		Cohort 1: 50.5 (8.9) Cohort 2: 47.2 (10.2)	Cohort 1: 50.5 (8.3) Cohort 2: 51.1 (9.7)	Cohort 1: 48.7 (8.8) Cohort 2: 49.0 (9.4)
Gavigan et al. 2020^b^ [23]				37.8 (34.0–40.8)	63.0 (58.7–67.9)	63.3 (60.3–66.9)			59.2 (54.3–63.0)				
Heisler et al. 2020 [24]		^m^										53.6 (8.9)	50.9 (9.1)
Hitchon et al. 2020 [25]												≥ 60^ h^ 24.0%	≥ 60^ h^ 22.7%
Hwang et al. 2020 [26]		45.6 (8.9)		46.6 (9.8)	51.1 (10.5)	52.2 (9.9)		45.7 (8.9)					45.3 (8.5)
Katz et al. 2020 [27]						56.3 (95)							
Liew et al. 2020 [28]												54.0 (9.0)	
Bingham et al. 2019 [30, 31]	Bingham et al. 2019 [30]		^m^	38.7 (9.4)	58.0 (11.6)^c^ 58.0 (11.5)^d^ 58.6 (11.6)^e^				53.9 (10.0)		44.8 (10.3)^d^	52.9 (10.1)	51.5 (10.5)**§§**
Crins et al. 2019 [32, 33]	Crins et al. 2019 [32]					53.6 (9.8)	56.7 (5.1)						
Izadi et al. 2019 [34]				40.1 (10.7)									
Mahmoud et al. 2019 [35]				37.6 (6.9)	59.3 (5.9)	61.1 (5.5)		53.6 (5.5)	54.6 (3.7)			59.9 (6.0)	57.7 (8.3)
Wohlfahrt et al. 2019 [36]		41.4 (7.3)^k^ 48.0 (8.2)^l^			56.8 (8.6)^c^	60.6 (7.3)	59.2 (4.7)	51.5 (6.0)	55.2 (9.8)^j^ 55.2 (8.5)			54.3 (8.8)	50.8 (9.7)
Bartlett et al. 2018 [30, 38]	Bartlett et al. cohort 1 [38]				Cohort 1: 54.8 (13.6)^d^ 54.6 (11.2)^e^								
	Bartlett et al. cohort 2 [38]				Cohort 2: 65.6 (8.1)^d^ 66.0 (7.8)^e^								
	Bartlett et al. cohort 3 [38]				Cohort 3: 53.2 (9.9)^d^ 55.3 (10.3)^e^								
Katz et al. 2018 [39]					59.3 (6.7)	60.9 (7.3)							
Bacalao et al. 2017 [41]				42.3 (5.9)	56.4 (9.2)	57.6 (7.5)							51.4 (8.4)
Katz et al. 2017 [42]			^m^	42.0 (9.1)	53.6 (11.0)	56.4 (9.4)			51.2 (9.3)	48.9 (9.7)		48.3 (9.2)	47.8 (8.7)
Wahl et al. 2017 [43]				40.2 (10.5)									
Askew et al. 2016 [44–47]	Article 1: Askew et al. 2016 [44]^a^ Article 2: Cella et al. 2015 [45] Article 3: Beaumont et al. 2021 [46] Article 4: Schalet et al. 2016 [47]^a^			Article 3: 40.7 (9.0) Article 4: 40.7 (39.9–41.5)	Article 2: 53.8 (8.8)^b^ Article 3: 53.7 (8.8)^g^	Article 1: 55.3 (54.6–56.0) Article 2: 55.2 (8.6)							
Fries et al. 2011 [52–54]	Fries et al. 2011 [53]				33.9 (22.0)^f^ 30.6 (21.0)^g^								

*APS* Ability to Participate in Social Roles and Activities; *axSpA* axial spondyloarthritis; *GH* Global Health; *Pain BH* Pain Behavior; *Pain IT* Pain Intensity; *Pain IF* Pain Interference; *P-29* PROMIS-29; *RA* rheumatoid arthritis; *SD* standard deviation

^a^Mean (95% Confidence Interval)

^b^Median (Interquartile range)

^c^4-items

^d^7-items

^e^8-items

^f^10-items

^g^20-items

^h^Outcome was not a T-score but a percentage of the population of a certain T-score

^i^PROMIS Sleep Disturbance unless stated otherwise

^j^PROMIS Sleep-Related Impairment

^k^PROMIS Physical Global Health

^l^PROMIS Mental Global Health

^m^ General Questionnaire was used, but only sub scores were reported

We contacted the authors of one study (18) as the reported score differed considerably from other reported scores (*T*-score PROMIS Ability to Participate in Social Roles) with the authors confirming its accuracy. For PROMIS measures where a higher score denotes better health, the mean *T*-scores were > 50 in only one of the 24 reported scores, reflecting the overall poorer health status of people with RA and axSpA. For PROMIS measures where a lower score indicates better health, the mean *T*-scores were < 50 in six of the 67 reported scores.

There were four PROMIS measures of which actual *T*-scores were reported in 10 or more articles: PROMIS Physical function: range mean 30.6–46.6, PROMIS Fatigue: range 51.1–66.0, PROMIS Depression: range 45.3–57.7, and PROMIS Pain Interference: range 52.2–65.8, overall indicating poor health.

## Discussion

This systematic literature review on the use of PROMIS measures in clinical studies in RA and axSpA patients identified 29 studies described in 40 articles. In total, two general health and 13 domain-specific PROMIS measures were used, with the PROMIS Pain interference, Fatigue, and Physical function and Depression being the measures that were most often reported. Overall, there was considerable variety concerning the versions of PROMIS measures that were used.

The 29 included studies were published from 2011 up to 2022, with relatively more articles published in recent years. As the total number of publications on clinical studies in RA and axSpA has also grown markedly, it remains to be ascertained whether the proportion of studies using PROMIS measures as outcome measures increased with time. Overall, the total number of identified studies using PROMIS measures is quite small as compared to the wealth of clinical studies in inflammatory arthritis published in the past two decades.

Regarding the nature of the PROMIS measures that were identified, most of the measures cover dimensions as described in the International Classification of Functioning, Disability and Health (ICF) Core Sets (Comprehensive and Brief) for Rheumatoid Arthritis and for Ankylosing Spondylitis [56, 57]. Similarly, the full range of measures is in line with the Outcome Measures in Rheumatoid Arthritis Clinical Trials (OMERACT) recommendations for outcome assessment in RA and axSpA patients in clinical trials [58, 59]. Both the ICF Core sets and OMERACT recommendations include health domains rather than specific measurement instruments, such as PROMIS measures. Specific measures are included in the more recently developed ICHOM core set for inflammatory arthritis, which particularly advocates the use of PROMIS measures, i.e., the PROMIS General Health, PROMIS Pain Interference, PROMIS Physical Function and PROMIS Fatigue measures [13]. In line with the ICHOM core set, we found that these were the PROMIS measures that were most often used. However, there was also substantial use of other PROMIS measures that were not recommended by ICHOM, such as the PROMIS Sleep Disturbance, PROMIS Abilities to Participate in Social Roles and Activities, PROMIS Depression and PROMIS Anxiety. Although not advocated by ICHOM, they do concern the domains as proposed by the OMERACT recommendations and the ICF core sets. It is unclear so far if the use of measures covering areas such as sleep indicates that the content of some established core sets must be revised. Moreover, the use of PROMIS measures also depends on the research question to be answered. Hence some studies warrant the use of not recommended PROMIS measures and within such studies the recommended PROMIS measures may be less relevant.

With respect to the actual scores of the PROMIS measures, the *T*-scores extracted from 21 studies were generally in line with the expectation that patients with RA and axSpA have a worse health status than the general population. There were, however, some exceptions where the *T*-scores indicated better health than expected, namely in the Depression and Abilities to Participate in Social Roles and Activities. Overall, the number of *T*-scores available per PROMIS measure was low, and often different versions of an instrument were used. Of note is that we observed considerable variation regarding the versions of specific PROMIS measures that were used. Although this could in part be explained by the launch of updates, there was also quite some variation regarding the number of items and the naming. It remains to be established if comparisons of scores where different versions of one measure have been used are valid. Therefore, taking the latter into account as well as the variation in the number of items and the naming of the PROMIS measures, we could not conduct subgroup analyses. Hence, conclusions on the level of *T*-scores for RA and axSpA patients cannot be drawn.

This study had some limitations that need to be addressed. First, as a result of the large diversity of the included studies in terms of follow-up moments, presentation of the data, and inclusion criteria, we did not yet review the data on psychometric properties of PROMIS measures according to the COnsensus-based Standards for the selection of health Measurement INstruments (COSMIN) guidelines. Second, the large variability between studies also hampered the further comparison between populations and studies in terms of a meta-analysis. Third, the limited amount of four studies reporting on axSpA patients (3 studies reported solely on axSpA, one study reported on RA and axSpA patients) compared to the 25 studies solely on RA patients which hampered the interpretation for the axSpA patient group. Subsequently, we were unable to compare these two groups together and we displayed the individual data and analyzed the total data of the RA patients and axSpA patients combined. Finally, the possible overrepresentation of the use of certain PROMIS measures as a result of studies that were based on similar populations. Some studies showed overlap with others but were considered as a separate study since the data were not exactly the same in terms of the sample sizes and general patient characteristics.

Nevertheless, the broad eligibility allowed the inclusion of most of the relevant literature, thereby presenting a fairly complete picture of the use of PROMIS measures in clinical research in inflammatory arthritis. The conduct of the study according to the PRISMA recommendations supports the accuracy and validity of the work.

In conclusion, currently, PROMIS measures are not often used in clinical studies in patients with RA and with axSpA. Within the studies that did use them considerable variety regarding the different PROMIS measures used as well as the specific versions of each instrument was present. As expected, the PROMIS measure outcomes depicted the overall impaired health outcomes in RA and axSpA populations. In future research, to facilitate comparisons across studies, more standardization regarding the use of PROMIS measures in clinical studies in RA and axSpA is needed.

## Supplementary Information

Below is the link to the electronic supplementary material.

Supplementary material 1 (DOC 173.5 kb)

## Data Availability

The datasets and/or analyses of the study are available from the corresponding author on reasonable request.
