# Supplementary material for: The use of PROMIS measures in clinical studies in patients with inflammatory arthritis: a systematic review
Source: Qual Life Res. 2023 Apr 27;32(10):2731–49. doi: 10.1007/s11136-023-03422-0 (PMC10474175; doi:10.1007/s11136-023-03422-0)
Supplement: Supplementary file 1 — Supplementary material 1 (DOC 173.5 kb) [file 11136_2023_3422_MOESM1_ESM.doc]

**Supplement 1** - Search strategies

**PubMed**

(("Inflammatory Arthritis"[tw] OR "Arthritis"[Mesh:NoExp] OR "Arthritis, Rheumatoid"[Mesh] OR "Rheumatoid Arthritis"[tw] OR "RA"[tw] OR "Caplan Syndrome"[tw] OR "Felty Syndrome"[tw] OR "Rheumatoid Nodule"[tw] OR "Rheumatoid Vasculitis"[tw] OR "Sjogren's Syndrome"[tw] OR "Sjoegren's Syndrome"[tw] OR "Sjogren Syndrome"[tw] OR "Sjoegren Syndrome"[tw] OR "Sjogrens Syndrome"[tw] OR "Sjoegrens Syndrome"[tw] OR "Sjogren's Syndrome"[tw] OR "Adult-Onset Still's Disease"[tw] OR "Adult-Onset Stills Disease"[tw] OR "Axial Spondyloarthritis"[Mesh] OR "axial spondylarthritis"[tw] OR "axial spondylarthrit*"[tw] OR "axial spondyloarthritis"[tw] OR "axial spondyloarthrit*"[tw] OR "AxSpA"[tw] OR "Ankylosing Spondylarthrit*"[tw] OR "Ankylosing Spondylarthritis"[tw] OR "Ankylosing Spondylitis"[tw] OR "Ankylosing Spondyl*"[tw] OR "Ankylosing Spondyloarthrit*"[tw] OR "Ankylosing Spondyloarthritis"[tw] OR "Bechterew Disease"[tw] OR "Bechterews Disease"[tw] OR "Bechterew's Disease"[tw] OR "Marie Struempell Disease"[tw] OR "Marie-Struempell Disease"[tw] OR "Rheumatoid Spondylitis"[tw] OR "Spondyloarthritis Ankylopoietica"[tw] OR "Spondylarthritis"[Mesh] OR "Spondylarthritis"[tw] OR "Spondylarthrit*"[tw] OR "Spondylarthropathies"[tw] OR "Spondylarthropathy"[tw] OR "Spondylarthr*"[tw] OR "Sacroiliitis"[mesh] OR "Sacroiliitis"[tw] OR "Sacroiliit*"[tw]) AND ("PROMIS"[tw] OR "PROMIS10"[tw] OR "NIHPROMIS"[tw] OR "PROMISPI"[tw] OR "PROMISPF"[tw] OR "PROMISGH"[tw] OR "PROMISSF"[tw] OR "Patient Reported Outcome Measurement Information System"[tw] OR "patient reported outcome measurement system"[tw] OR "patient reported outcome measurement"[tw] OR "Patient Reported Outcomes Measurement Information System"[tw] OR "patient reported outcomes measurement system"[tw] OR "patient reported outcomes measurement"[tw] OR (("Patient-Reported Outcome*"[tw] OR "Patientreported Outcome*"[tw] OR "PROM"[tw] OR "PROMS"[tw]) AND ("Information System*"[tw] OR "measurement system*"[tw] OR "item bank*"[tw] OR "itembank*"[tw]))))

**MEDLINE via OVID**

(("Inflammatory Arthritis".mp OR "Arthritis"/ OR exp "Arthritis, Rheumatoid"/ OR "Rheumatoid Arthritis".mp OR "RA".mp OR "Caplan Syndrome".mp OR "Felty Syndrome".mp OR "Rheumatoid Nodule".mp OR "Rheumatoid Vasculitis".mp OR "Sjogren's Syndrome".mp OR "Sjoegren's Syndrome".mp OR "Sjogren Syndrome".mp OR "Sjoegren Syndrome".mp OR "Sjogrens Syndrome".mp OR "Sjoegrens Syndrome".mp OR "Sjogren's Syndrome".mp OR "Adult-Onset Still's Disease".mp OR "Adult-Onset Stills Disease".mp OR exp "Axial Spondyloarthritis"/ OR "axial spondylarthritis".mp OR "axial spondylarthrit*".mp OR "axial spondyloarthritis".mp OR "axial spondyloarthrit*".mp OR "AxSpA".mp OR "Ankylosing Spondylarthrit*".mp OR "Ankylosing Spondylarthritis".mp OR "Ankylosing Spondylitis".mp OR "Ankylosing Spondyl*".mp OR "Ankylosing Spondyloarthrit*".mp OR "Ankylosing Spondyloarthritis".mp OR "Bechterew Disease".mp OR "Bechterews Disease".mp OR "Bechterew's Disease".mp OR "Marie Struempell Disease".mp OR "Marie-Struempell Disease".mp OR "Rheumatoid Spondylitis".mp OR "Spondyloarthritis Ankylopoietica".mp OR exp "Spondylarthritis"/ OR "Spondylarthritis".mp OR "Spondylarthrit*".mp OR "Spondylarthropathies".mp OR "Spondylarthropathy".mp OR "Spondylarthr*".mp OR exp "Sacroiliitis"/ OR "Sacroiliitis".mp OR "Sacroiliit*".mp) AND ("PROMIS".mp OR "PROMIS10".mp OR "NIHPROMIS".mp OR "PROMISPI".mp OR "PROMISPF".mp OR "PROMISGH".mp OR "PROMISSF".mp OR "Patient Reported Outcome Measurement Information System".mp OR "patient reported outcome measurement system".mp OR "patient reported outcome measurement".mp OR "Patient Reported Outcomes Measurement Information System".mp OR "patient reported outcomes measurement system".mp OR "patient reported outcomes measurement".mp OR (("Patient-Reported Outcome*".mp OR "Patientreported Outcome*".mp OR "PROM".mp OR "PROMS".mp) AND ("Information System*".mp OR "measurement system*".mp OR "item bank*".mp OR "itembank*".mp))))

**Embase via OVID**

(("Inflammatory Arthritis".mp OR exp "Rheumatoid Arthritis"/ OR "Rheumatoid Arthritis".mp OR "RA".mp OR "Caplan Syndrome".mp OR "Felty Syndrome".mp OR "Rheumatoid Nodule".mp OR "Rheumatoid Vasculitis".mp OR "Sjogren's Syndrome".mp OR "Sjoegren's Syndrome".mp OR "Sjogren Syndrome".mp OR "Sjoegren Syndrome".mp OR "Sjogrens Syndrome".mp OR "Sjoegrens Syndrome".mp OR "Sjogren's Syndrome".mp OR "Adult-Onset Still's Disease".mp OR "Adult-Onset Stills Disease".mp OR "Spondyloarthritis"/ OR "axial spondylarthritis".mp OR "axial spondylarthrit*".mp OR "axial spondyloarthritis".mp OR "axial spondyloarthrit*".mp OR "AxSpA".mp OR "ankylosing spondylitis"/ OR "Ankylosing Spondylarthrit*".mp OR "Ankylosing Spondylarthritis".mp OR "Ankylosing Spondylit*".mp OR "Ankylosing Spondyloarthrit*".mp OR "Ankylosing Spondyloarthritis".mp OR "Bechterew Disease".mp OR "Bechterews Disease".mp OR "Bechterew's Disease".mp OR "Marie Struempell Disease".mp OR "Marie-Struempell Disease".mp OR "Rheumatoid Spondylitis".mp OR "Spondyloarthritis Ankylopoietica".mp OR "Spondylarthritis".mp OR "Spondylarthr*".mp OR "Spondylarthropathy"/ OR "Spondylarthropathies".mp OR "Spondylarthropathy".mp OR "Spondylarthr*".mp OR exp "Sacroiliitis"/ OR "Sacroiliitis".mp OR "Sacroiliit*".mp) AND ("PROMIS".mp OR "PROMIS10".mp OR "NIHPROMIS".mp OR "PROMISPI".mp OR "PROMISPF".mp OR "PROMISGH".mp OR "PROMISSF".mp OR "Patient Reported Outcome Measurement Information System".mp OR "patient reported outcome measurement system".mp OR "patient reported outcome measurement".mp OR "Patient Reported Outcomes Measurement Information System".mp OR "patient reported outcomes measurement system".mp OR "patient reported outcomes measurement".mp OR (("Patient-Reported Outcome*".mp OR "Patientreported Outcome*".mp OR "PROM".mp OR "PROMS".mp) AND ("Information System*".mp OR "measurement system*".mp OR "item bank*".mp OR "itembank*".mp)))) NOT (conference review or conference abstract).pt

**Web of Science**

(TS=("Inflammatory Arthritis" OR "Rheumatoid Arthritis" OR "Rheumatoid Arthritis" OR "RA" OR "Caplan Syndrome" OR "Felty Syndrome" OR "Rheumatoid Nodule" OR "Rheumatoid Vasculitis" OR "Sjogren's Syndrome" OR "Sjoegren's Syndrome" OR "Sjogren Syndrome" OR "Sjoegren Syndrome" OR "Sjogrens Syndrome" OR "Sjoegrens Syndrome" OR "Sjogren's Syndrome" OR "Adult-Onset Still's Disease" OR "Adult-Onset Stills Disease" OR "Spondyloarthritis" OR "axial spondylarthritis" OR "axial spondylarthrit*" OR "axial spondyloarthritis" OR "axial spondyloarthrit*" OR "AxSpA" OR "ankylosing spondylitis" OR "Ankylosing Spondylarthrit*" OR "Ankylosing Spondylarthritis" OR "Ankylosing Spondylit*" OR "Ankylosing Spondyloarthrit*" OR "Ankylosing Spondyloarthritis" OR "Bechterew Disease" OR "Bechterews Disease" OR "Bechterew's Disease" OR "Marie Struempell Disease" OR "Marie-Struempell Disease" OR "Rheumatoid Spondylitis" OR "Spondyloarthritis Ankylopoietica" OR "Spondylarthritis" OR "Spondylarthrit*" OR "Spondylarthr*" OR "Spondylarthropathy" OR "Spondylarthropathies" OR "Spondylarthropathy" OR "Spondylarthr*" OR "Sacroiliitis" OR "Sacroiliitis" OR "Sacroiliit*") AND TS=("PROMIS" OR "PROMIS10" OR "NIHPROMIS" OR "PROMISPI" OR "PROMISPF" OR "PROMISGH" OR "PROMISSF" OR "Patient Reported Outcome Measurement Information System" OR "patient reported outcome measurement system" OR "patient reported outcome measurement" OR "Patient Reported Outcomes Measurement Information System" OR "patient reported outcomes measurement system" OR "patient reported outcomes measurement" OR (("Patient-Reported Outcome*" OR "Patientreported Outcome*" OR "PROM" OR "PROMS") AND ("Information System*" OR "measurement system*" OR "item bank*" OR "itembank*")))) NOT DT=(meeting abstract)

**Cochrane Library**

(("Inflammatory Arthritis" OR "Rheumatoid Arthritis" OR "Rheumatoid Arthritis" OR "RA" OR "Caplan Syndrome" OR "Felty Syndrome" OR "Rheumatoid Nodule" OR "Rheumatoid Vasculitis" OR "Sjogren's Syndrome" OR "Sjoegren's Syndrome" OR "Sjogren Syndrome" OR "Sjoegren Syndrome" OR "Sjogrens Syndrome" OR "Sjoegrens Syndrome" OR "Sjogren's Syndrome" OR "Adult Onset Still's Disease" OR "Adult Onset Stills Disease" OR "Spondyloarthritis" OR "axial spondylarthritis" OR "axial spondylarthrit*" OR "axial spondyloarthritis" OR "axial spondyloarthrit*" OR "AxSpA" OR "ankylosing spondylitis" OR "Ankylosing Spondylarthrit*" OR "Ankylosing Spondylarthritis" OR "Ankylosing Spondylit*" OR "Ankylosing Spondyloarthrit*" OR "Ankylosing Spondyloarthritis" OR "Bechterew Disease" OR "Bechterews Disease" OR "Bechterew's Disease" OR "Marie Struempell Disease" OR "Marie Struempell Disease" OR "Rheumatoid Spondylitis" OR "Spondyloarthritis Ankylopoietica" OR "Spondylarthritis" OR "Spondylarthrit*" OR "Spondylarthr*" OR "Spondylarthropathy" OR "Spondylarthropathies" OR "Spondylarthropathy" OR "Spondylarthr*" OR "Sacroiliitis" OR "Sacroiliitis" OR "Sacroiliit*") AND ("PROMIS" OR "PROMIS10" OR "NIHPROMIS" OR "PROMISPI" OR "PROMISPF" OR "PROMISGH" OR "PROMISSF" OR "Patient Reported Outcome Measurement Information System" OR "patient reported outcome measurement system" OR "Patient Reported Outcomes Measurement Information System" OR "patient reported outcomes measurement system")):ti,ab,kw

**Emcare via OVID**

(("Inflammatory Arthritis".mp OR exp "Rheumatoid Arthritis"/ OR "Rheumatoid Arthritis".mp OR "RA".mp OR "Caplan Syndrome".mp OR "Felty Syndrome".mp OR "Rheumatoid Nodule".mp OR "Rheumatoid Vasculitis".mp OR "Sjogren's Syndrome".mp OR "Sjoegren's Syndrome".mp OR "Sjogren Syndrome".mp OR "Sjoegren Syndrome".mp OR "Sjogrens Syndrome".mp OR "Sjoegrens Syndrome".mp OR "Sjogren's Syndrome".mp OR "Adult-Onset Still's Disease".mp OR "Adult-Onset Stills Disease".mp OR "Spondyloarthritis"/ OR "axial spondylarthritis".mp OR "axial spondylarthrit*".mp OR "axial spondyloarthritis".mp OR "axial spondyloarthrit*".mp OR "AxSpA".mp OR "ankylosing spondylitis"/ OR "Ankylosing Spondylarthrit*".mp OR "Ankylosing Spondylarthritis".mp OR "Ankylosing Spondylit*".mp OR "Ankylosing Spondyloarthrit*".mp OR "Ankylosing Spondyloarthritis".mp OR "Bechterew Disease".mp OR "Bechterews Disease".mp OR "Bechterew's Disease".mp OR "Marie Struempell Disease".mp OR "Marie-Struempell Disease".mp OR "Rheumatoid Spondylitis".mp OR "Spondyloarthritis Ankylopoietica".mp OR "Spondylarthritis".mp OR "Spondylarthr*".mp OR "Spondylarthropathy"/ OR "Spondylarthropathies".mp OR "Spondylarthropathy".mp OR "Spondylarthr*".mp OR exp "Sacroiliitis"/ OR "Sacroiliitis".mp OR "Sacroiliit*".mp) AND ("PROMIS".mp OR "PROMIS10".mp OR "NIHPROMIS".mp OR "PROMISPI".mp OR "PROMISPF".mp OR "PROMISGH".mp OR "PROMISSF".mp OR "Patient Reported Outcome Measurement Information System".mp OR "patient reported outcome measurement system".mp OR "patient reported outcome measurement".mp OR "Patient Reported Outcomes Measurement Information System".mp OR "patient reported outcomes measurement system".mp OR "patient reported outcomes measurement".mp OR (("Patient-Reported Outcome*".mp OR "Patientreported Outcome*".mp OR "PROM".mp OR "PROMS".mp) AND ("Information System*".mp OR "measurement system*".mp OR "item bank*".mp OR "itembank*".mp))))

**PsycINFO via EbscoHOST**

TX(("Inflammatory Arthritis" OR "Rheumatoid Arthritis" OR "Rheumatoid Arthritis" OR "RA" OR "Caplan Syndrome" OR "Felty Syndrome" OR "Rheumatoid Nodule" OR "Rheumatoid Vasculitis" OR "Sjogren's Syndrome" OR "Sjoegren's Syndrome" OR "Sjogren Syndrome" OR "Sjoegren Syndrome" OR "Sjogrens Syndrome" OR "Sjoegrens Syndrome" OR "Sjogren's Syndrome" OR "Adult Onset Still's Disease" OR "Adult Onset Stills Disease" OR "Spondyloarthritis" OR "axial spondylarthritis" OR "axial spondylarthrit*" OR "axial spondyloarthritis" OR "axial spondyloarthrit*" OR "AxSpA" OR "ankylosing spondylitis" OR "Ankylosing Spondylarthrit*" OR "Ankylosing Spondylarthritis" OR "Ankylosing Spondylit*" OR "Ankylosing Spondyloarthrit*" OR "Ankylosing Spondyloarthritis" OR "Bechterew Disease" OR "Bechterews Disease" OR "Bechterew's Disease" OR "Marie Struempell Disease" OR "Marie Struempell Disease" OR "Rheumatoid Spondylitis" OR "Spondyloarthritis Ankylopoietica" OR "Spondylarthritis" OR "Spondylarthrit*" OR "Spondylarthr*" OR "Spondylarthropathy" OR "Spondylarthropathies" OR "Spondylarthropathy" OR "Spondylarthr*" OR "Sacroiliitis" OR "Sacroiliitis" OR "Sacroiliit*") AND ("PROMIS" OR "PROMIS10" OR "NIHPROMIS" OR "PROMISPI" OR "PROMISPF" OR "PROMISGH" OR "PROMISSF" OR "Patient Reported Outcome Measurement Information System" OR "patient reported outcome measurement system" OR "patient reported outcome measurement" OR "Patient Reported Outcomes Measurement Information System" OR "patient reported outcomes measurement system" OR "patient reported outcomes measurement" OR (("Patient-Reported Outcome*" OR "Patientreported Outcome*" OR "PROM" OR "PROMS") AND ("Information System*" OR "measurement system*" OR "item bank*" OR "itembank*"))))

**Academic Search Premier via EbscoHOST**

(TI("Inflammatory Arthritis" OR "Rheumatoid Arthritis" OR "Rheumatoid Arthritis" OR "RA" OR "Caplan Syndrome" OR "Felty Syndrome" OR "Rheumatoid Nodule" OR "Rheumatoid Vasculitis" OR "Sjogren's Syndrome" OR "Sjoegren's Syndrome" OR "Sjogren Syndrome" OR "Sjoegren Syndrome" OR "Sjogrens Syndrome" OR "Sjoegrens Syndrome" OR "Sjogren's Syndrome" OR "Adult Onset Still's Disease" OR "Adult Onset Stills Disease" OR "Spondyloarthritis" OR "axial spondylarthritis" OR "axial spondylarthrit*" OR "axial spondyloarthritis" OR "axial spondyloarthrit*" OR "AxSpA" OR "ankylosing spondylitis" OR "Ankylosing Spondylarthrit*" OR "Ankylosing Spondylarthritis" OR "Ankylosing Spondylit*" OR "Ankylosing Spondyloarthrit*" OR "Ankylosing Spondyloarthritis" OR "Bechterew Disease" OR "Bechterews Disease" OR "Bechterew's Disease" OR "Marie Struempell Disease" OR "Marie Struempell Disease" OR "Rheumatoid Spondylitis" OR "Spondyloarthritis Ankylopoietica" OR "Spondylarthritis" OR "Spondylarthrit*" OR "Spondylarthr*" OR "Spondylarthropathy" OR "Spondylarthropathies" OR "Spondylarthropathy" OR "Spondylarthr*" OR "Sacroiliitis" OR "Sacroiliitis" OR "Sacroiliit*") AND TX("PROMIS" OR "PROMIS10" OR "NIHPROMIS" OR "PROMISPI" OR "PROMISPF" OR "PROMISGH" OR "PROMISSF" OR "Patient Reported Outcome Measurement Information System" OR "patient reported outcome measurement system" OR "Patient Reported Outcomes Measurement Information System" OR "patient reported outcomes measurement system")) **OR** (SU("Inflammatory Arthritis" OR "Rheumatoid Arthritis" OR "Rheumatoid Arthritis" OR "RA" OR "Caplan Syndrome" OR "Felty Syndrome" OR "Rheumatoid Nodule" OR "Rheumatoid Vasculitis" OR "Sjogren's Syndrome" OR "Sjoegren's Syndrome" OR "Sjogren Syndrome" OR "Sjoegren Syndrome" OR "Sjogrens Syndrome" OR "Sjoegrens Syndrome" OR "Sjogren's Syndrome" OR "Adult Onset Still's Disease" OR "Adult Onset Stills Disease" OR "Spondyloarthritis" OR "axial spondylarthritis" OR "axial spondylarthrit*" OR "axial spondyloarthritis" OR "axial spondyloarthrit*" OR "AxSpA" OR "ankylosing spondylitis" OR "Ankylosing Spondylarthrit*" OR "Ankylosing Spondylarthritis" OR "Ankylosing Spondylit*" OR "Ankylosing Spondyloarthrit*" OR "Ankylosing Spondyloarthritis" OR "Bechterew Disease" OR "Bechterews Disease" OR "Bechterew's Disease" OR "Marie Struempell Disease" OR "Marie Struempell Disease" OR "Rheumatoid Spondylitis" OR "Spondyloarthritis Ankylopoietica" OR "Spondylarthritis" OR "Spondylarthrit*" OR "Spondylarthr*" OR "Spondylarthropathy" OR "Spondylarthropathies" OR "Spondylarthropathy" OR "Spondylarthr*" OR "Sacroiliitis" OR "Sacroiliitis" OR "Sacroiliit*") AND TI("PROMIS" OR "PROMIS10" OR "NIHPROMIS" OR "PROMISPI" OR "PROMISPF" OR "PROMISGH" OR "PROMISSF" OR "Patient Reported Outcome Measurement Information System" OR "patient reported outcome measurement system" OR "Patient Reported Outcomes Measurement Information System" OR "patient reported outcomes measurement system")) **OR** (AB("Inflammatory Arthritis" OR "Rheumatoid Arthritis" OR "Rheumatoid Arthritis" OR "RA" OR "Caplan Syndrome" OR "Felty Syndrome" OR "Rheumatoid Nodule" OR "Rheumatoid Vasculitis" OR "Sjogren's Syndrome" OR "Sjoegren's Syndrome" OR "Sjogren Syndrome" OR "Sjoegren Syndrome" OR "Sjogrens Syndrome" OR "Sjoegrens Syndrome" OR "Sjogren's Syndrome" OR "Adult Onset Still's Disease" OR "Adult Onset Stills Disease" OR "Spondyloarthritis" OR "axial spondylarthritis" OR "axial spondylarthrit*" OR "axial spondyloarthritis" OR "axial spondyloarthrit*" OR "AxSpA" OR "ankylosing spondylitis" OR "Ankylosing Spondylarthrit*" OR "Ankylosing Spondylarthritis" OR "Ankylosing Spondylit*" OR "Ankylosing Spondyloarthrit*" OR "Ankylosing Spondyloarthritis" OR "Bechterew Disease" OR "Bechterews Disease" OR "Bechterew's Disease" OR "Marie Struempell Disease" OR "Marie Struempell Disease" OR "Rheumatoid Spondylitis" OR "Spondyloarthritis Ankylopoietica" OR "Spondylarthritis" OR "Spondylarthrit*" OR "Spondylarthr*" OR "Spondylarthropathy" OR "Spondylarthropathies" OR "Spondylarthropathy" OR "Spondylarthr*" OR "Sacroiliitis" OR "Sacroiliitis" OR "Sacroiliit*") AND TI("PROMIS" OR "PROMIS10" OR "NIHPROMIS" OR "PROMISPI" OR "PROMISPF" OR "PROMISGH" OR "PROMISSF" OR "Patient Reported Outcome Measurement Information System" OR "patient reported outcome measurement system" OR "Patient Reported Outcomes Measurement Information System" OR "patient reported outcomes measurement system")) **OR** (KW("Inflammatory Arthritis" OR "Rheumatoid Arthritis" OR "Rheumatoid Arthritis" OR "RA" OR "Caplan Syndrome" OR "Felty Syndrome" OR "Rheumatoid Nodule" OR "Rheumatoid Vasculitis" OR "Sjogren's Syndrome" OR "Sjoegren's Syndrome" OR "Sjogren Syndrome" OR "Sjoegren Syndrome" OR "Sjogrens Syndrome" OR "Sjoegrens Syndrome" OR "Sjogren's Syndrome" OR "Adult Onset Still's Disease" OR "Adult Onset Stills Disease" OR "Spondyloarthritis" OR "axial spondylarthritis" OR "axial spondylarthrit*" OR "axial spondyloarthritis" OR "axial spondyloarthrit*" OR "AxSpA" OR "ankylosing spondylitis" OR "Ankylosing Spondylarthrit*" OR "Ankylosing Spondylarthritis" OR "Ankylosing Spondylit*" OR "Ankylosing Spondyloarthrit*" OR "Ankylosing Spondyloarthritis" OR "Bechterew Disease" OR "Bechterews Disease" OR "Bechterew's Disease" OR "Marie Struempell Disease" OR "Marie Struempell Disease" OR "Rheumatoid Spondylitis" OR "Spondyloarthritis Ankylopoietica" OR "Spondylarthritis" OR "Spondylarthrit*" OR "Spondylarthr*" OR "Spondylarthropathy" OR "Spondylarthropathies" OR "Spondylarthropathy" OR "Spondylarthr*" OR "Sacroiliitis" OR "Sacroiliitis" OR "Sacroiliit*") AND TI("PROMIS" OR "PROMIS10" OR "NIHPROMIS" OR "PROMISPI" OR "PROMISPF" OR "PROMISGH" OR "PROMISSF" OR "Patient Reported Outcome Measurement Information System" OR "patient reported outcome measurement system" OR "Patient Reported Outcomes Measurement Information System" OR "patient reported outcomes measurement system"))

**Google Scholar**

Five query variations (first 10 items selected)

"Patient Reported Outcome Measurement Information"|"patient reported outcome measurement system"|"Patient Reported Outcomes Measurement Information"|"Patient Reported Outcomes Measurement Information" "Inflammatory Arthritis"

(first 10 items selected)

"Patient Reported Outcome Measurement Information"|"patient reported outcome measurement system"|"Patient Reported Outcomes Measurement Information"|"Patient Reported Outcomes Measurement Information" "Rheumatoid Arthritis"

(first 10 items selected)

"Patient Reported Outcome Measurement Information"|"patient reported outcome measurement system"|"Patient Reported Outcomes Measurement Information"|"Patient Reported Outcomes Measurement Information" "axial spondylarthritis"|"AxSpA"

(first 10 items selected)

"Patient Reported Outcome Measurement Information"|"patient reported outcome measurement system"|"Patient Reported Outcomes Measurement Information"|"Patient Reported Outcomes Measurement Information" "ankylosing spondylitis"|"Sacroiliitis"

(first 10 items selected)

"PROMIS" "Inflammatory Arthritis"|"Rheumatoid Arthritis"|"axial spondylarthritis"|"AxSpA"|"ankylosing spondylitis"|"Sacroiliitis"

(first 40 items selected)
